# 2-(*tert*-Butoxy­carbonyl­amino)-6-(1,3-dioxo-1*H*-2,3-dihydro­benzo[*de*]isoquinolin-2-yl)hexa­noic acid

**DOI:** 10.1107/S1600536810014935

**Published:** 2010-04-28

**Authors:** Hoong-Kun Fun, Jia Hao Goh, Zhenjun Qiu, Yan Zhang

**Affiliations:** a X-ray Crystallography Unit, School of Physics, Universiti Sains Malaysia, 11800 USM, Penang, Malaysia; b School of Chemistry and Chemical Engineering, Key Laboratory of Analytical Chemistry for Life Science, Ministry of Education of China, Nanjing University, Nanjing 210093, People’s Republic of China

## Abstract

In the title naphthalimide derivative, C_23_H_26_N_2_O_6_, the 1,8-naphthalimide system is essentially planar [maximum deviation = 0.0456 (16) Å]. A characteristic pattern of alternating long and short C—C bond lengths is observed in the 1,8-naphthalimide unit. The mean planes through the methyl carbamate and acetic acid groups form dihedral angles of 42.30 (9) and 61.59 (9)°, respectively, with the 1,8-naphthalimide plane. In the crystal structure, inter­molecular O—H⋯O and C—H⋯O hydrogen bonds link neighbouring mol­ecules, forming *R*_2_^2^(9) hydrogen-bond ring motifs. These rings are further inter­connected by inter­molecular N—H⋯O and C—H⋯O hydrogen bonds into a three-dimensional supra­molecular network.

## Related literature

For general background to and applications of 1,8-naphthal­imide derivatives, see: Abraham *et al.* (2004 ▶); Hung *et al.* (2005 ▶); Le *et al.* (2000 ▶); Pogozelski & Tullius (1998 ▶); Saito *et al.* (1995**a* ▶,b* ▶). For details of hydrogen-bond motifs, see: Bernstein *et al.* (1995 ▶). For related structures, see: Clark & Hall (1989 ▶); Zarychta *et al.* (2003 ▶). For bond-length data, see: Allen *et al.* (1987 ▶). For the stability of the temperature controller used for the data collection, see: Cosier & Glazer (1986 ▶).
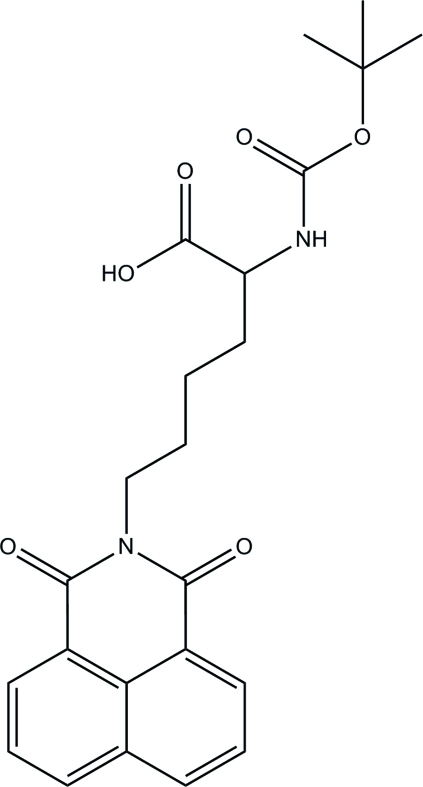


## Experimental

### 

#### Crystal data

C_23_H_26_N_2_O_6_
*M* *_r_* = 426.46
Monoclinic, 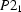

*a* = 5.1681 (13) Å
*b* = 15.427 (4) Å
*c* = 13.426 (3) Å
β = 91.491 (5)°
*V* = 1070.1 (5) Å^3^
*Z* = 2
Mo *K*α radiation
μ = 0.10 mm^−1^
*T* = 100 K
0.22 × 0.20 × 0.18 mm

#### Data collection

Bruker SMART APEX DUO CCD area-detector diffractometer
Absorption correction: multi-scan (*SADABS*; Bruker, 2009 ▶) *T*_min_ = 0.980, *T*_max_ = 0.983
10037 measured reflections
2540 independent reflections
2320 reflections with *I* > 2σ(*I*)
*R*_int_ = 0.035

#### Refinement

*R*[*F* ^2^ > 2σ(*F* ^2^)] = 0.032
*wR*(*F* ^2^) = 0.083
*S* = 1.04
2540 reflections
291 parameters
1 restraint
H atoms treated by a mixture of independent and constrained refinement
Δρ_max_ = 0.24 e Å^−3^
Δρ_min_ = −0.20 e Å^−3^

### 

Data collection: *APEX2* (Bruker, 2009 ▶); cell refinement: *SAINT* (Bruker, 2009 ▶); data reduction: *SAINT*; program(s) used to solve structure: *SHELXTL* (Sheldrick, 2008 ▶); program(s) used to refine structure: *SHELXTL*; molecular graphics: *SHELXTL*; software used to prepare material for publication: *SHELXTL* and *PLATON* (Spek, 2009 ▶).

## Supplementary Material

Crystal structure: contains datablocks global, I. DOI: 10.1107/S1600536810014935/sj2760sup1.cif

Structure factors: contains datablocks I. DOI: 10.1107/S1600536810014935/sj2760Isup2.hkl

Additional supplementary materials:  crystallographic information; 3D view; checkCIF report

## Figures and Tables

**Table 1:** Hydrogen-bond geometry (Å, °)

*D*—H⋯*A*	*D*—H	H⋯*A*	*D*⋯*A*	*D*—H⋯*A*
N2—H1*N*2⋯O1^i^	0.86 (3)	2.17 (3)	3.008 (2)	166 (3)
O6—H1*O*6⋯O3^ii^	0.87 (3)	1.84 (3)	2.695 (2)	166 (3)
C3—H3*A*⋯O5^iii^	0.93	2.41	3.331 (3)	169
C7—H7*A*⋯O5^iv^	0.93	2.45	3.183 (3)	136

Symmetry codes: (i) 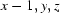; (ii) 
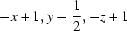
; (iii) 
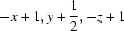
; (iv) 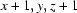.

**Fig. 1.**
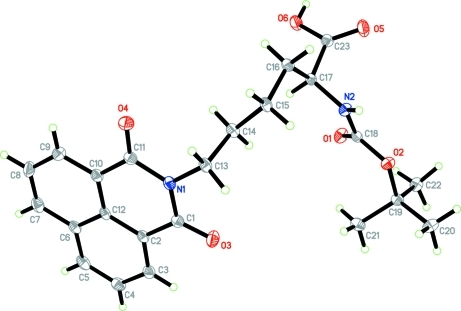
The molecular structure of the title compound, showing 50% probability displacement ellipsoids for non-H atoms and the atom-numbering scheme.

**Fig. 2.**
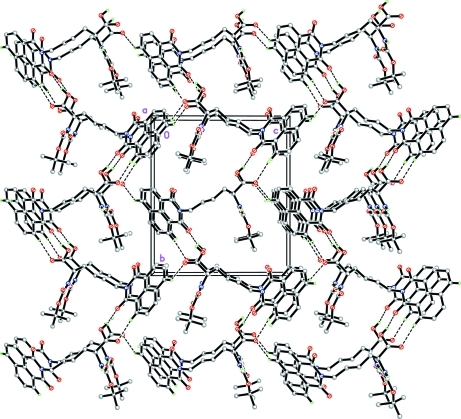
The crystal structure of the title compound, viewed along the a axis, showing the three-dimensional supramolecular network. H atoms not involved in intermolecular interactions (dashed lines) have been omitted for clarity.

## supplementary crystallographic information

### Comment

1,8-Naphthalimides are useful molecular probes for their unique luminescence and transient properties (Pogozelski & Tullius, 1998). They have a diversity of reactivity towards biological substrates (Pogozelski & Tullius, 1998). 1,8-Naphthalimide derivatives have attracted significant attention due to not only their participation in photoinduced electron transfer (PET) processes (Le *et al.*, 2000; Abraham *et al.*, 2004), but also to their applications in the fields of biology and medicine (Saito *et al.*, 1995*a*). Some 1,8-naphthalimide derivatives have been reported to inhibit virulence regulation in Vibrio cholerae by inhibiting the transcriptional regulator ToxT (Hung *et al.*, 2005). Other 1,8-naphthalimide derivatives have also been used in the photosensitized one-electron oxidation of DNA through the PET process (Saito *et al.*, 1995*b*). In view of the importance of the 1,8-naphthalimide derivatives, the title compound was obtained and this paper reports its crystal structure.

In the title compound, the 1,8-naphthalimide moiety (N1/C1-C12/O3/O4) is essentially planar, with maximum deviation of 0.0456 (16) Å at atom O3. The characteristic alternating pattern of C—C bond lengths is observed in the naphthalimide ring system, specifically, C2—C3, C4—C5, C7—C8 and C9—C10 bond lengths are shorter than the expected aromatic C—C bond length [average value of 1.373 (3) Å], whereas all the other bond lengths in the aromatic rings are longer than expected value [average value of 1.412 (3) Å]. This characteristic pattern of bond length variation has been reported previously in other *N*-substituted naphthalimide structures (Clark & Hall, 1989; Zarychta *et al.*, 2003). All other bond lengths (Allen *et al.*, 1987) and angles are within normal range. The plane through the 1,8-naphthalimide ring system forms dihedral angles of 42.30 (9) and 61.59 (9)°, respectively, with those through the methyl carbamate (C17/N2/C18/O1/O2) and acetic acid (C17/C23/O5/O6) groups.

In the crystal structure, intermolecular O6—H1O6···O3 and C3—H3A···O5 hydrogen bonds (Table 1) link neighbouring molecules into *R*_2_^2^(9) hydrogen bond ring motifs (Bernstein *et al.*, 1995). These ring motifs are further interconnected by intermolecular N2—H1N2···O1 and C7—H7A···O5 hydrogen bonds (Table 1) into a three-dimensional supramolecular network.

### Experimental

The title compound was derived from the reaction between 1,8-naphthalic anhydride and α-N-Boc-L-Lysine in anhydrous dimethylformamide. Removal of the solvent under reduced pressure followed by silica gel chromatography gave the title compound. X-ray quality single crystals of the tile compound were obtained from slow evaporation of a methanol/ether solution (1:2, v:v).

### Refinement

Atoms H1N2 and H1O6 were located from difference Fourier map and allowed to refine freely. All other hydrogen atoms were placed in their calculated positions, with C—H = 0.93 – 0.97 Å, and refined using a riding model, with *U*_iso_ = 1.2 or 1.5 *U*_eq_(C). A rotating group model was used for the methyl groups. In the absence of significant anomalous dispersion, 2210 Friedel pairs were merged for the final refinement.

### Crystal data

**Table:**

C_23_H_26_N_2_O_6_	*F*(000) = 452
*M**_r_* = 426.46	*D*_x_ = 1.324 Mg m^−^^3^
Monoclinic, *P*2_1_	Mo *K*α radiation, λ = 0.71073 Å
Hall symbol: P 2yb	Cell parameters from 3461 reflections
*a* = 5.1681 (13) Å	θ = 3.0–31.4°
*b* = 15.427 (4) Å	µ = 0.10 mm^−^^1^
*c* = 13.426 (3) Å	*T* = 100 K
β = 91.491 (5)°	Block, colourless
*V* = 1070.1 (5) Å^3^	0.22 × 0.20 × 0.18 mm
*Z* = 2	

### Data collection

**Table:**

Bruker SMART APEX DUO CCD area-detector diffractometer	2540 independent reflections
Radiation source: fine-focus sealed tube	2320 reflections with *I* > 2σ(*I*)
graphite	*R*_int_ = 0.035
φ and ω scans	θ_max_ = 27.5°, θ_min_ = 1.5°
Absorption correction: multi-scan (*SADABS*; Bruker, 2009)	*h* = −6→6
*T*_min_ = 0.980, *T*_max_ = 0.983	*k* = −20→19
10037 measured reflections	*l* = −17→17

### Refinement

**Table:**

Refinement on *F*^2^	Primary atom site location: structure-invariant direct methods
Least-squares matrix: full	Secondary atom site location: difference Fourier map
*R*[*F*^2^ > 2σ(*F*^2^)] = 0.032	Hydrogen site location: inferred from neighbouring sites
*wR*(*F*^2^) = 0.083	H atoms treated by a mixture of independent and constrained refinement
*S* = 1.04	*w* = 1/[σ^2^(*F*_o_^2^) + (0.0471*P*)^2^ + 0.1369*P*] where *P* = (*F*_o_^2^ + 2*F*_c_^2^)/3
2540 reflections	(Δ/σ)_max_ < 0.001
291 parameters	Δρ_max_ = 0.24 e Å^−^^3^
1 restraint	Δρ_min_ = −0.20 e Å^−^^3^

### Special details

**Table:**

Experimental. The crystal was placed in the cold stream of an Oxford Cryosystems Cobra open-flow nitrogen cryostat (Cosier & Glazer, 1986) operating at 100.0 (1)K.
Geometry. All esds (except the esd in the dihedral angle between two l.s. planes) are estimated using the full covariance matrix. The cell esds are taken into account individually in the estimation of esds in distances, angles and torsion angles; correlations between esds in cell parameters are only used when they are defined by crystal symmetry. An approximate (isotropic) treatment of cell esds is used for estimating esds involving l.s. planes.
Refinement. Refinement of F^2^ against ALL reflections. The weighted R-factor wR and goodness of fit S are based on F^2^, conventional R-factors R are based on F, with F set to zero for negative F^2^. The threshold expression of F^2^ > 2sigma(F^2^) is used only for calculating R-factors(gt) etc. and is not relevant to the choice of reflections for refinement. R-factors based on F^2^ are statistically about twice as large as those based on F, and R- factors based on ALL data will be even larger.

### Fractional atomic coordinates and isotropic or equivalent isotropic displacement parameters (Å^2^)

**Table:**

	*x*	*y*	*z*	*U*_iso_*/*U*_eq_	
O1	0.5844 (3)	0.09878 (10)	0.34654 (12)	0.0196 (3)	
O2	0.2687 (3)	0.19431 (9)	0.29685 (12)	0.0162 (3)	
O3	0.4054 (3)	0.22297 (10)	0.73839 (12)	0.0223 (4)	
O4	0.1240 (3)	−0.03594 (11)	0.85133 (11)	0.0220 (3)	
O5	0.2044 (3)	−0.08287 (11)	0.24623 (12)	0.0276 (4)	
O6	0.4014 (3)	−0.14959 (11)	0.37506 (12)	0.0237 (4)	
N1	0.2618 (3)	0.09498 (12)	0.79736 (13)	0.0154 (4)	
N2	0.1589 (3)	0.06803 (11)	0.36137 (13)	0.0145 (4)	
C1	0.4270 (4)	0.16611 (13)	0.80155 (16)	0.0154 (4)	
C2	0.6260 (4)	0.16820 (14)	0.88308 (16)	0.0152 (4)	
C3	0.7899 (4)	0.23814 (14)	0.89240 (16)	0.0179 (4)	
H3A	0.7728	0.2848	0.8488	0.021*	
C4	0.9847 (4)	0.23901 (15)	0.96853 (17)	0.0205 (5)	
H4A	1.0956	0.2863	0.9748	0.025*	
C5	1.0105 (4)	0.17055 (15)	1.03290 (17)	0.0200 (5)	
H5A	1.1410	0.1716	1.0819	0.024*	
C6	0.8428 (4)	0.09837 (14)	1.02641 (15)	0.0163 (4)	
C7	0.8582 (4)	0.02823 (15)	1.09386 (17)	0.0213 (5)	
H7A	0.9857	0.0281	1.1440	0.026*	
C8	0.6875 (4)	−0.03981 (16)	1.08636 (17)	0.0224 (5)	
H8A	0.6989	−0.0853	1.1317	0.027*	
C9	0.4951 (4)	−0.04085 (15)	1.01006 (17)	0.0198 (4)	
H9A	0.3801	−0.0871	1.0051	0.024*	
C10	0.4767 (4)	0.02633 (14)	0.94287 (15)	0.0163 (4)	
C11	0.2741 (4)	0.02366 (13)	0.86285 (16)	0.0156 (4)	
C12	0.6468 (4)	0.09776 (14)	0.95005 (15)	0.0144 (4)	
C13	0.0601 (4)	0.09147 (14)	0.71759 (15)	0.0161 (4)	
H13A	0.0173	0.1500	0.6966	0.019*	
H13B	−0.0949	0.0654	0.7436	0.019*	
C14	0.1460 (4)	0.03956 (14)	0.62756 (16)	0.0165 (4)	
H14A	0.2178	−0.0154	0.6498	0.020*	
H14B	0.2805	0.0711	0.5940	0.020*	
C15	−0.0811 (4)	0.02293 (14)	0.55430 (15)	0.0154 (4)	
H15A	−0.2266	0.0013	0.5911	0.018*	
H15B	−0.1330	0.0776	0.5241	0.018*	
C16	−0.0208 (4)	−0.04160 (14)	0.47149 (15)	0.0150 (4)	
H16A	−0.1728	−0.0478	0.4282	0.018*	
H16B	0.0158	−0.0977	0.5012	0.018*	
C17	0.2092 (4)	−0.01458 (13)	0.40848 (15)	0.0133 (4)	
H17A	0.3611	−0.0081	0.4531	0.016*	
C18	0.3572 (4)	0.11921 (13)	0.33550 (15)	0.0145 (4)	
C19	0.4489 (4)	0.26771 (13)	0.28446 (17)	0.0159 (4)	
C20	0.2696 (4)	0.34116 (15)	0.25217 (18)	0.0209 (5)	
H20A	0.1394	0.3491	0.3011	0.031*	
H20B	0.1883	0.3271	0.1891	0.031*	
H20C	0.3678	0.3936	0.2460	0.031*	
C21	0.5825 (5)	0.28823 (16)	0.38377 (18)	0.0249 (5)	
H21A	0.7006	0.2423	0.4013	0.037*	
H21B	0.4552	0.2936	0.4342	0.037*	
H21C	0.6761	0.3417	0.3785	0.037*	
C22	0.6367 (4)	0.24803 (15)	0.20234 (17)	0.0189 (4)	
H22A	0.7565	0.2042	0.2247	0.028*	
H22B	0.7299	0.2997	0.1861	0.028*	
H22C	0.5424	0.2278	0.1444	0.028*	
C23	0.2680 (4)	−0.08485 (14)	0.33260 (15)	0.0155 (4)	
H1N2	0.002 (6)	0.0845 (19)	0.351 (2)	0.029 (7)*	
H1O6	0.451 (6)	−0.186 (2)	0.330 (2)	0.034 (8)*	

### Atomic displacement parameters (Å^2^)

**Table:**

	*U*^11^	*U*^22^	*U*^33^	*U*^12^	*U*^13^	*U*^23^
O1	0.0104 (6)	0.0218 (8)	0.0265 (9)	0.0011 (6)	−0.0011 (6)	0.0055 (6)
O2	0.0114 (6)	0.0147 (7)	0.0225 (8)	−0.0011 (6)	−0.0014 (6)	0.0018 (6)
O3	0.0218 (8)	0.0244 (9)	0.0205 (8)	−0.0045 (6)	−0.0046 (6)	0.0072 (6)
O4	0.0233 (7)	0.0202 (8)	0.0222 (8)	−0.0050 (7)	−0.0024 (6)	−0.0004 (7)
O5	0.0419 (10)	0.0250 (9)	0.0155 (8)	0.0130 (8)	−0.0066 (7)	−0.0022 (7)
O6	0.0347 (9)	0.0188 (8)	0.0172 (8)	0.0124 (7)	−0.0058 (7)	−0.0041 (7)
N1	0.0143 (7)	0.0182 (9)	0.0136 (9)	−0.0006 (7)	−0.0013 (6)	−0.0004 (7)
N2	0.0090 (7)	0.0146 (9)	0.0198 (9)	0.0016 (6)	−0.0008 (6)	0.0019 (7)
C1	0.0146 (9)	0.0164 (10)	0.0152 (10)	0.0010 (8)	0.0021 (7)	−0.0013 (8)
C2	0.0150 (9)	0.0179 (10)	0.0128 (10)	0.0016 (8)	0.0013 (7)	−0.0026 (8)
C3	0.0189 (9)	0.0198 (11)	0.0152 (11)	−0.0006 (8)	0.0036 (8)	−0.0005 (8)
C4	0.0176 (10)	0.0230 (11)	0.0209 (12)	−0.0050 (9)	0.0016 (8)	−0.0047 (9)
C5	0.0141 (9)	0.0288 (12)	0.0171 (11)	0.0015 (9)	−0.0021 (8)	−0.0060 (9)
C6	0.0142 (9)	0.0207 (10)	0.0140 (10)	0.0029 (8)	0.0016 (7)	−0.0042 (8)
C7	0.0210 (10)	0.0271 (12)	0.0157 (11)	0.0067 (9)	−0.0018 (8)	−0.0012 (9)
C8	0.0287 (11)	0.0226 (11)	0.0158 (11)	0.0066 (10)	0.0006 (9)	0.0052 (9)
C9	0.0216 (10)	0.0176 (10)	0.0201 (11)	−0.0014 (9)	0.0015 (8)	0.0018 (9)
C10	0.0172 (10)	0.0182 (11)	0.0136 (10)	0.0019 (8)	0.0013 (8)	−0.0019 (8)
C11	0.0146 (9)	0.0165 (10)	0.0157 (10)	−0.0001 (8)	0.0015 (8)	−0.0019 (8)
C12	0.0145 (8)	0.0184 (10)	0.0105 (9)	0.0023 (8)	0.0018 (7)	−0.0020 (8)
C13	0.0135 (8)	0.0202 (10)	0.0144 (10)	0.0020 (8)	−0.0020 (7)	−0.0013 (8)
C14	0.0138 (9)	0.0202 (10)	0.0155 (10)	0.0024 (8)	−0.0006 (7)	−0.0025 (8)
C15	0.0127 (9)	0.0204 (10)	0.0129 (10)	0.0010 (8)	−0.0025 (7)	−0.0007 (8)
C16	0.0124 (8)	0.0174 (10)	0.0150 (10)	−0.0017 (8)	−0.0021 (7)	−0.0013 (8)
C17	0.0123 (8)	0.0140 (10)	0.0133 (10)	0.0009 (7)	−0.0020 (7)	0.0000 (7)
C18	0.0151 (9)	0.0150 (10)	0.0133 (10)	0.0009 (8)	−0.0015 (7)	−0.0022 (7)
C19	0.0133 (9)	0.0156 (10)	0.0185 (11)	−0.0031 (8)	−0.0021 (8)	0.0001 (8)
C20	0.0178 (10)	0.0150 (10)	0.0300 (13)	0.0001 (8)	0.0031 (9)	0.0027 (9)
C21	0.0283 (11)	0.0225 (12)	0.0235 (12)	−0.0052 (10)	−0.0062 (9)	−0.0037 (10)
C22	0.0134 (9)	0.0213 (11)	0.0220 (12)	0.0001 (8)	0.0003 (8)	0.0025 (9)
C23	0.0138 (8)	0.0162 (10)	0.0163 (10)	0.0012 (8)	−0.0015 (7)	−0.0008 (8)

### Geometric parameters (Å, °)

**Table:**

O1—C18	1.221 (2)	C9—H9A	0.9300
O2—C18	1.345 (2)	C10—C12	1.411 (3)
O2—C19	1.478 (2)	C10—C11	1.481 (3)
O3—C1	1.223 (3)	C13—C14	1.525 (3)
O4—C11	1.210 (3)	C13—H13A	0.9700
O5—C23	1.197 (3)	C13—H13B	0.9700
O6—C23	1.333 (3)	C14—C15	1.533 (3)
O6—H1O6	0.87 (3)	C14—H14A	0.9700
N1—C1	1.391 (3)	C14—H14B	0.9700
N1—C11	1.409 (3)	C15—C16	1.531 (3)
N1—C13	1.476 (2)	C15—H15A	0.9700
N2—C18	1.346 (3)	C15—H15B	0.9700
N2—C17	1.443 (3)	C16—C17	1.535 (3)
N2—H1N2	0.86 (3)	C16—H16A	0.9700
C1—C2	1.483 (3)	C16—H16B	0.9700
C2—C3	1.375 (3)	C17—C23	1.524 (3)
C2—C12	1.413 (3)	C17—H17A	0.9800
C3—C4	1.416 (3)	C19—C22	1.518 (3)
C3—H3A	0.9300	C19—C21	1.519 (3)
C4—C5	1.369 (3)	C19—C20	1.520 (3)
C4—H4A	0.9300	C20—H20A	0.9600
C5—C6	1.412 (3)	C20—H20B	0.9600
C5—H5A	0.9300	C20—H20C	0.9600
C6—C7	1.412 (3)	C21—H21A	0.9600
C6—C12	1.422 (3)	C21—H21B	0.9600
C7—C8	1.373 (3)	C21—H21C	0.9600
C7—H7A	0.9300	C22—H22A	0.9600
C8—C9	1.409 (3)	C22—H22B	0.9600
C8—H8A	0.9300	C22—H22C	0.9600
C9—C10	1.376 (3)		
			
C18—O2—C19	119.64 (15)	C15—C14—H14A	109.4
C23—O6—H1O6	110 (2)	C13—C14—H14B	109.4
C1—N1—C11	124.98 (17)	C15—C14—H14B	109.4
C1—N1—C13	118.66 (17)	H14A—C14—H14B	108.0
C11—N1—C13	116.34 (17)	C16—C15—C14	114.07 (17)
C18—N2—C17	120.08 (16)	C16—C15—H15A	108.7
C18—N2—H1N2	120.3 (19)	C14—C15—H15A	108.7
C17—N2—H1N2	119.6 (19)	C16—C15—H15B	108.7
O3—C1—N1	119.54 (19)	C14—C15—H15B	108.7
O3—C1—C2	123.03 (19)	H15A—C15—H15B	107.6
N1—C1—C2	117.42 (18)	C15—C16—C17	113.53 (17)
C3—C2—C12	120.60 (19)	C15—C16—H16A	108.9
C3—C2—C1	119.87 (19)	C17—C16—H16A	108.9
C12—C2—C1	119.53 (18)	C15—C16—H16B	108.9
C2—C3—C4	119.9 (2)	C17—C16—H16B	108.9
C2—C3—H3A	120.1	H16A—C16—H16B	107.7
C4—C3—H3A	120.1	N2—C17—C23	111.83 (17)
C5—C4—C3	120.3 (2)	N2—C17—C16	110.37 (16)
C5—C4—H4A	119.9	C23—C17—C16	110.26 (17)
C3—C4—H4A	119.9	N2—C17—H17A	108.1
C4—C5—C6	121.33 (19)	C23—C17—H17A	108.1
C4—C5—H5A	119.3	C16—C17—H17A	108.1
C6—C5—H5A	119.3	O1—C18—O2	125.83 (19)
C7—C6—C5	122.71 (19)	O1—C18—N2	123.60 (19)
C7—C6—C12	119.0 (2)	O2—C18—N2	110.57 (16)
C5—C6—C12	118.3 (2)	O2—C19—C22	110.18 (17)
C8—C7—C6	120.9 (2)	O2—C19—C21	109.53 (18)
C8—C7—H7A	119.6	C22—C19—C21	113.24 (18)
C6—C7—H7A	119.6	O2—C19—C20	102.83 (15)
C7—C8—C9	120.1 (2)	C22—C19—C20	109.78 (18)
C7—C8—H8A	119.9	C21—C19—C20	110.79 (18)
C9—C8—H8A	119.9	C19—C20—H20A	109.5
C10—C9—C8	120.2 (2)	C19—C20—H20B	109.5
C10—C9—H9A	119.9	H20A—C20—H20B	109.5
C8—C9—H9A	119.9	C19—C20—H20C	109.5
C9—C10—C12	120.7 (2)	H20A—C20—H20C	109.5
C9—C10—C11	119.34 (19)	H20B—C20—H20C	109.5
C12—C10—C11	119.91 (19)	C19—C21—H21A	109.5
O4—C11—N1	119.69 (18)	C19—C21—H21B	109.5
O4—C11—C10	123.53 (19)	H21A—C21—H21B	109.5
N1—C11—C10	116.78 (17)	C19—C21—H21C	109.5
C10—C12—C2	121.35 (18)	H21A—C21—H21C	109.5
C10—C12—C6	119.01 (19)	H21B—C21—H21C	109.5
C2—C12—C6	119.64 (19)	C19—C22—H22A	109.5
N1—C13—C14	112.33 (16)	C19—C22—H22B	109.5
N1—C13—H13A	109.1	H22A—C22—H22B	109.5
C14—C13—H13A	109.1	C19—C22—H22C	109.5
N1—C13—H13B	109.1	H22A—C22—H22C	109.5
C14—C13—H13B	109.1	H22B—C22—H22C	109.5
H13A—C13—H13B	107.9	O5—C23—O6	124.0 (2)
C13—C14—C15	111.26 (16)	O5—C23—C17	125.09 (19)
C13—C14—H14A	109.4	O6—C23—C17	110.91 (17)
			
C11—N1—C1—O3	177.12 (19)	C9—C10—C12—C6	1.7 (3)
C13—N1—C1—O3	−1.5 (3)	C11—C10—C12—C6	−178.95 (18)
C11—N1—C1—C2	−2.0 (3)	C3—C2—C12—C10	178.64 (19)
C13—N1—C1—C2	179.44 (17)	C1—C2—C12—C10	−1.9 (3)
O3—C1—C2—C3	2.7 (3)	C3—C2—C12—C6	−1.4 (3)
N1—C1—C2—C3	−178.21 (19)	C1—C2—C12—C6	178.03 (18)
O3—C1—C2—C12	−176.7 (2)	C7—C6—C12—C10	−1.2 (3)
N1—C1—C2—C12	2.3 (3)	C5—C6—C12—C10	−179.66 (19)
C12—C2—C3—C4	1.2 (3)	C7—C6—C12—C2	178.89 (19)
C1—C2—C3—C4	−178.20 (19)	C5—C6—C12—C2	0.4 (3)
C2—C3—C4—C5	0.0 (3)	C1—N1—C13—C14	95.2 (2)
C3—C4—C5—C6	−1.0 (3)	C11—N1—C13—C14	−83.5 (2)
C4—C5—C6—C7	−177.6 (2)	N1—C13—C14—C15	169.74 (17)
C4—C5—C6—C12	0.8 (3)	C13—C14—C15—C16	−170.05 (18)
C5—C6—C7—C8	178.5 (2)	C14—C15—C16—C17	−57.8 (2)
C12—C6—C7—C8	0.0 (3)	C18—N2—C17—C23	−82.3 (2)
C6—C7—C8—C9	0.6 (3)	C18—N2—C17—C16	154.59 (17)
C7—C8—C9—C10	−0.2 (3)	C15—C16—C17—N2	−59.8 (2)
C8—C9—C10—C12	−1.0 (3)	C15—C16—C17—C23	176.17 (16)
C8—C9—C10—C11	179.6 (2)	C19—O2—C18—O1	−13.6 (3)
C1—N1—C11—O4	−178.4 (2)	C19—O2—C18—N2	166.25 (17)
C13—N1—C11—O4	0.2 (3)	C17—N2—C18—O1	2.5 (3)
C1—N1—C11—C10	1.1 (3)	C17—N2—C18—O2	−177.36 (17)
C13—N1—C11—C10	179.68 (17)	C18—O2—C19—C22	69.9 (2)
C9—C10—C11—O4	−1.6 (3)	C18—O2—C19—C21	−55.3 (2)
C12—C10—C11—O4	179.0 (2)	C18—O2—C19—C20	−173.11 (18)
C9—C10—C11—N1	178.90 (19)	N2—C17—C23—O5	−22.3 (3)
C12—C10—C11—N1	−0.5 (3)	C16—C17—C23—O5	100.9 (2)
C9—C10—C12—C2	−178.4 (2)	N2—C17—C23—O6	157.54 (16)
C11—C10—C12—C2	1.0 (3)	C16—C17—C23—O6	−79.3 (2)

### Hydrogen-bond geometry (Å, °)

**Table:**

*D*—H···*A*	*D*—H	H···*A*	*D*···*A*	*D*—H···*A*
N2—H1N2···O1^i^	0.86 (3)	2.17 (3)	3.008 (2)	166 (3)
O6—H1O6···O3^ii^	0.87 (3)	1.84 (3)	2.695 (2)	166 (3)
C3—H3A···O5^iii^	0.93	2.41	3.331 (3)	169
C7—H7A···O5^iv^	0.93	2.45	3.183 (3)	136

Symmetry codes: (i) *x*−1, *y*, *z*; (ii) −*x*+1, *y*−1/2, −*z*+1; (iii) −*x*+1, *y*+1/2, −*z*+1; (iv) *x*+1, *y*, *z*+1.
